# Influence of Asphaltene Modification on Structure of P3HT/Asphaltene Blends: Molecular Dynamics Simulations

**DOI:** 10.3390/nano12162867

**Published:** 2022-08-20

**Authors:** Natalia Borzdun, Artyom Glova, Sergey Larin, Sergey Lyulin

**Affiliations:** 1 Institute of Macromolecular Compounds, Russian Academy of Sciences, Bolshoi pr. 31 (V.O.), 199004 St. Petersburg, Russia; 2 Faculty of Physics, St. Petersburg State University, Ulyanovskaya str. 1–3, Peterhof, 198504 St. Petersburg, Russia

**Keywords:** poly(3-hexylthiophene), asphaltenes, bulk heterojunction blend morphology, molecular dynamics simulations

## Abstract

Further development and commercialization of bulk heterojunction (BHJ) solar cells require the search for novel low-cost materials. The present study addresses the relations between the asphaltenes’ chemical structure and the morphology of the poly(3-hexylthiohene) (P3HT)/asphaltene blends as potential materials for the design of BHJ solar cells. By means of all-atom molecular dynamics simulations, the formation of heterophase morphology is observed for the P3HT-based blends with carboxyl-containing asphaltenes, as well as the aggregation of the asphaltenes into highly ordered stacks. Although the π–π interactions between the polyaromatic cores of the asphaltenes in solutions are sufficient for the molecules to aggregate into ordered stacks, in a blend with a conjugated polymer, additional stabilizing factors are required, such as hydrogen bonding between carboxyl groups. It is found that the asphaltenes’ aliphatic side groups may improve significantly the miscibility between the polymer and the asphaltenes, thereby preventing the formation of heterophase morphology. The results also demonstrate that the carboxyl-containing asphaltenes/P3HT ratio should be at least 1:1, as a decrease in concentration of the asphaltenes leads to the folding of the polymer chains, lower ordering in the polymer phase and the destruction of the interpenetrating 3D structure formed by P3HT and the asphaltene phases. Overall, the results of the present study for the first time reveal the aggregation behavior of the asphaltenes of varying chemical structures in P3HT, as well the influence of their presence and concentration on the polymer phase structure and blend morphology, paving the way for future development of BHJ solar cells based on the conjugated polymer/asphaltene blends.

## 1. Introduction

Nowadays, there is an active search for new acceptor materials for bulk heterojunction (BHJ) solar cells, both to increase the power conversion efficiency and stability of the devices and to reduce their production costs [1,2]. While conjugated polymers receive significant attention as donor materials, graphene-like small molecules are of considerable interest as acceptors due to their ability to form stacked structures via π–π interactions, which provides enhanced charge transfer in acceptor regions [3]. Asphaltenes are natural graphene-like small compounds that occur in nature as a mixture of molecules of diverse architecture and can be characterized by the presence of a polycyclic aromatic core, aliphatic side groups and various types of heteroatoms [4]. As an abandoned by-product of deep oil refining, asphaltenes are orders of magnitude cheaper than analogous synthetic small carbon nanoparticles [5,6,7]. Although several studies have recently suggested the use of asphaltenes in various electronic applications [8,9,10,11] and even demonstrated the first prototypes of devices [12,13], the literature data on the possibility of using conjugated polymer/asphaltene blends in organic electronics remain scarce [14].

Indeed, various types of organic semiconductors were utilized as acceptors in polymer solar cells. The most famous type of such molecules are fullerene-based derivatives—mainly phenyl-C_61_-butyric acid methyl ester (PCBM) and phenyl-C_71_-butyric acid methyl ester (PC_71_BM) [15,16]. However, fullerenes have significant drawbacks, namely narrow and weak absorption in the visible region and difficulty of tuning the absorption and electronic energy levels [17,18]. In recent years, various non-fullerene acceptors (NFA) based on fused ring core demonstrated great advantages over fullerene acceptors. Among them, one can mention the IDTBR [19] and ITIC [20] families of NFA that show good performance in polymer solar cells. The asphaltenes have some similarities in the chemical structure with many NFA; namely, they contain polycyclic conjugated core and aliphatic or functional side groups [17]. This allows us to suggest that asphaltenes could also be utilized as non-fullerene acceptors in polythiophene-based organic photovoltaic devices. At the same time, natural variations of asphaltene chemical structure could also improve the morphological stability and lifetime of organic photovoltaic devices, as it was shown that an increase in the number of photoactive components in the blend could stabilize the molecular arrangement and phase separation of components due to higher entropy of the mixture [21]. The increased morphological stability of asphaltene-containing photovoltaic devices could be of high importance for indoor use, which has an increasing importance nowadays [21,22,23].

As we have shown previously by means of density functional theory (DFT) calculations [24], the chemical modification of asphaltenes with carboxyl groups enables their energy levels to be fine tuned, thereby obtaining the required energy difference between the lowest unoccupied molecular orbitals (LUMO) of the acceptor and donor materials for efficient charge transfer.

In that work, we chose the well-established regioregular poly(3-hexylthiophene) (P3HT) as a model donor, as P3HT is widely studied by means of computer simulations [25,26,27] and as thiophene-based polymers are one of the best classic choices for use as electron donors in BHJ solar cells [28,29,30,31]. It should be noted that the other types of polythiophene donors developed recently, such as PM6, PBDB-T and others, could provide higher performance of organic solar cells than P3HT [17,21,28]. However, P3HT is widely used as a model polymer, as it has a rather simple chemical structure, and the effects related to the interaction of acceptor molecules either with the conjugated main chain or aliphatic side groups could be easily distinguished, especially in computer simulations. Thus, we used P3HT to understand the influence of the chemical structure of asphaltenes, i.e., various combinations of the aromatic core with aliphatic or functional groups, on the structure of donor/acceptor blends.

The molecular dynamics (MD) simulations of the carboxyl-containing asphaltenes/P3HT blend revealed the heterophase morphology of the studied blend, as well as the formation of extended stacks of the asphaltenes, all of the above being essential for efficient charge transfer in the active layer. We also observed a slight ordering of the P3HT backbones, possibly due to the impact of the addition of asphaltenes [24].

However, the exact reason for such structure formation remains unclear. A possible explanation for the formation of the heterophase morphology of the blend and the ordering of P3HT chains is in the specific interactions between the aromatic backbone of the polymer and the polyaromatic core of the studied carboxyl-containing asphaltenes. The presence of aliphatic side groups or polar carboxyl groups should affect the miscibility between the components of the mixture. In turn, the formation of stacks between asphaltene molecules might be influenced by both π-π stacking between the polyaromatic cores of the asphaltenes and the hydrogen bonding between the −COOH groups of the adjacent molecules [32].

To gain a better understanding of the factors governing the perspective morphological properties of the P3HT/carboxyl-containing asphaltene blend observed previously [15], in the present study, we test which type of interaction has the major contribution to the formation of ordered stacks of the carboxyl-containing asphaltenes. To this end, we perform MD simulations of P3HT-based blends containing model asphaltene molecules of the following three types: the model asphaltene molecule with aliphatic side groups proposed by Mullins [33], Li and Greenfield [34]; the model molecule with aliphatic chains cut off [35]; and another modification of the model molecule containing the carboxyl groups studied previously [24]. The asphaltene model molecule with aliphatic groups is expected to have high compatibility with P3HT due to the presence of both aromatic and aliphatic constituents in the chemical structure. Of greatest interest is the difference between the asphaltene with aliphatic groups cut off and the carboxyl-containing asphaltene. MD simulations of these two model asphaltenes mixed with P3HT might reveal the reason for the formation of the asphaltene aggregates of stacked geometry.

It should be noted that there are some complexities related to the structural organization of P3HT as a standalone phase, along with the impact on the formation of heterojunctions. The structure of P3HT, its solid-state organization and its polymorphs are at the core of a long-lasting debate. It is believed that P3HT could form two polymorphs [27,36,37,38,39]. Indeed, only one of its polymorphs has been characterized, although only partially, since the fractional coordinates have not been reported. That is the form II polymorph, as reported for the P3HT polymer chains of different molecular weights by Rahimi et al. [36]. Strong uncertainties exist regarding the form I, which is thought to be the mesophase present in bulk heterojunctions, generally obtained from spin coating processes and thus the most frequently occurring. No universally agreed structural model has been proposed so far in the literature for the form I structure. At the same time, it is rather difficult to obtain polymer crystallization in a molecular dynamics simulation even using long timescales. Thus, in our work, we concentrated only on the structure of P3HT blends with asphaltenes and ordering of P3HT backbone but not on the investigation of P3HT polymorphs.

It is well known that the blend composition, namely the ratio of the two components, impacts significantly on the microstructure of an active layer and, consequently, the device efficiency [40]. Therefore, a rational choice of the optimal concentration is required to ensure the formation of phase-separated regions of the two interpenetrating continuous phases. Thus, in this study, we performed simulations of the blends with varying amount of carboxyl-containing asphaltenes added into the mixture to establish the possible effect of the P3HT/asphaltenes ratio on the heterophase structure of the mixtures and the morphology of the polymer phase. Overall, the goal of the present study was to investigate the structure and concentration effects in the blends of P3HT with asphaltenes.

## 2. Models and Methods

**Figure 1:**
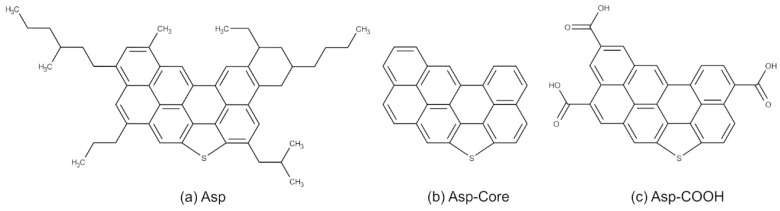
Chemical structures of the model asphaltene molecules with (**a**) aliphatic side groups proposed by Mullins [33], Li and Greenfield [34] (Asp), (**b**) aliphatic side groups cut-off (Asp-Core) [35] and (**c**) three carboxyl groups (Asp-COOH) [24].

Asphaltenes occur in nature as a mixture of molecules of different chemical structures. Although various complicated asphaltene architectures were used in MD simulations [7,41,42], in this study, we considered the following previously investigated and well-established types of model molecules (Figure 1): the asphaltene with aliphatic side groups proposed by Mullins [33], Li and Greenfield [34] (denoted as Asp in the text and figures); the same model molecule with side aliphatic chains cut off (Asp-Core) [35]; another modification of the Asp molecules - the carboxyl-containing asphaltene with three −COOH groups (Asp-COOH) [24]. The Asp molecule contains 7 aromatic rings with a single sulfur atom. The Asp-Core could be obtained in the experiment by means of cracking that leads to the cut-off of all aliphatic fragments, including the aliphatic ring. Structures similar to the Asp-COOH model molecule could be obtained by asphaltene treatment with oxidizing agents. Naturally, the number of carboxyl groups in such molecule could be different, and it could influence both the electronic properties of asphaltene molecules and their interaction with a polymer. However, it was shown that the Asp-COOH asphaltene with 3 carboxyl groups had the most appropriate electronic properties to be used as an acceptor molecule in organic solar cells [24] with P3HT used as donor, and therefore, it was chosen as a model carboxyl-functionalized asphaltene.

The MD simulations were performed using the GROMACS software package [43]. The general Amber force field (GAFF) [44] was used to describe bonded and non-bonded interactions in the systems. The atomic partial charges were calculated by the semi-empirical AM1-BCC method utilizing the ACPYPE tool [44,45]. Partial charges for all types of molecules investigated are provided in the Supporting Information. This force field was successfully implemented previously for the simulations of both asphaltenes [46,47,48,49] and polythiophenes [50,51,52,53,54]. In particular, the validations of P3HT and asphaltene models were performed in our previous studies [24,35,46].

We should note that there is a rather large number of force fields developed especially to model P3HT [55,56,57]. However, we prefer to use unmodified GAFF parameters, taking into account the following reasons. First, the studied systems are the mixtures of P3HT with other compounds, which were not specially parametrized in the above-mentioned force fields. Thus, in order to preserve the compatibility between the force field description of the system components, we use the unmodified GAFF both for P3HT and asphaltenes. At the same time, the special P3HT force fields are often optimized to reproduce the crystal structure of P3HT [56,57], which is not achieved in our simulation due to spatial restraints associated with the presence of aggregates formed by asphaltenes in the systems under study. Therefore, we could expect that using an unmodified GAFF parameter set will not have sufficient influence on the simulation results presented in the paper.

Three types of systems with the same asphaltenes/P3HT mass ratio were simulated. Namely, 27 P3HT chains consisting of *N_p_* = 40 repeating units each (27,054 atoms) were blended with (a) 254 Asp molecules (46,710 atoms in the system in total), (b) 504 Asp-Core (56,010 atoms) and (c) 368 Asp-COOH molecules (44,718 atoms). Thus, in these examined systems, the mass ratio of the components was approximately equal to 1:1. The chosen polymerization degree of P3HT corresponds to the polymer regime where P3HT properties slightly depend on the polymer molecular weight. Such polymerization degree is often used in the simulation of this polymer [31,58,59,60].

Initially, semi-coiled P3HT chains and asphaltene molecules for each system were randomly distributed in a large cubic box (with box dimensions of 25 nm in each direction, density of approx. 40 kg/m^3^) with periodic boundary conditions using the GROMACS *gmx insert-molecules* routine. Then, to achieve a condensed state, a 5 ns long compression procedure was carried out at a temperature *T* = 600 K and pressure *P* = 100 bar utilizing the Berendsen thermostat and barostat [61] with time constants of 0.1 and 0.5 ps, respectively. After this compression, the pressure was lowered to 1 bar, and an additional 5 ns long run was carried out. At this stage the system density achieve value close to the experimental one (approximately 900 kg/m^3^). Finally, the main 5 µs long simulation runs were performed at a temperature *T* = 600 K and pressure *P* = 1 bar with the Nose–Hoover thermostat and Parrinello–Rahman barostat with time constants of 1.0 and 5.0 ps, respectively [62,63,64]. The simulation time step was equal to 2 fs. The electrostatic interactions were handled by the particle mesh Ewald method [65]. Additional simulations of the Asp-COOH/P3HT blend were performed in comparison with Ref [24]. According to the time dependencies of the radius of gyration *R_g_* calculated for the polymer chains in the blends studied, the systems can be considered equilibrated after approximately 2.5 µs of simulations, as will be discussed in the Results and Discussion section below. The required equilibration time is also confirmed by the analysis of time dependencies of the number of asphaltene clusters in the considered systems (Supporting Information, Figure S5). Mean-squared displacements of the centers of mass of P3HT chains also show that approximately 1 µs of simulations is required for the molecules to move over a distance comparable to the characteristic size of a polymer coil, while the diffusion of asphaltenes occurs much faster (Supporting Information, Figure S6). Thus, the 2.5 µs long runs are sufficient to obtain equilibrated configurations of the studied systems. The remaining 2.5 µs of simulations were utilized to study the structural properties of the blends. After equilibration, the size of all systems considered was approximately 9 × 9 × 9 nm^3^. The systems’ dimensions obtained after equilibration are more than twice as large as the gyration radius of the P3HT chain and 5–7 times larger than the size of asphaltene molecules. The chosen length scales and the achieved time scales correspond to the state-of-the-art simulations employed in computational material science [66,67]. This allows us to be sure that the structures obtained in the simulations are not artifacts due to the finite size effect. Nevertheless, to increase system size further, one can use coarse-grained models, taking into account the chemical structures of all components of the systems. To develop such a model, the results of our atomistic simulations could be used.

Thermal annealing is commonly applied to P3HT-based blends to control two morphological effects: the enhancement of polymer crystallinity and the improvement of phase separation [68,69]. Thus, in this study, we performed simulations at a temperature of 600 K, which is slightly above the P3HT melting point (~500–515 K [70,71,72,73]), as transition temperatures in simulations are usually higher than that observed in the experiment [67,74]. Additionally, we should note that although the melting temperature depends slightly on the polymerization degree, the experimentally determined melt transition point for P3HT with *N_p_* = 42 is close enough to that of a polymer with much higher molecular weight [73]. This confirms that the polymerization degree of P3HT chosen in our study corresponds to the beginning of the polymer regime, and the results obtained in our simulation will not change sufficiently if the higher polymerization degree is taken into account.

To investigate the concentration effects on the morphology of the blends, we performed additional simulations of the most promising blends based on Asp-COOH with mass ratios asphaltenes/P3HT equal to 2:3 and 1:3 (245 and 123 asphaltene molecules, respectively). The equilibration and simulation procedures were similar to the ones described above.

## 3. Results and Discussion

### 3.1. Structure of Asphaltene Phase

The formation of the heterophase structure of donor/acceptor blends, as well as the aggregation of graphene-like acceptor molecules into stacks, is necessary for the efficient operation of BHJ solar cells on their basis [3,75,76]. Such structure formation is expected for the Asp-COOH/P3HT blend, as shown by our preliminary estimates [24]. However, the key factors responsible for the formation of such morphology remain uncovered. In the case of P3HT mixtures with all types of asphaltenes considered, the π–π-interactions between aromatic fragments of mixture components are present. However, in the case of Asp molecules, the additional interaction between aliphatic groups of asphaltene and aliphatic side chains of P3HT could influence the mixture morphology. Additionally, for the Asp-COOH molecules, hydrogen bonding could be formed, stabilizing the asphaltene aggregates. Thus, the present study aims at investigating the effect of the nature of the asphaltenes’ side groups and the corresponding types of interactions on the morphology of P3HT/asphaltene blends, as well as the concentration effects and their impact on the structure of the blends.

**Figure 2:**
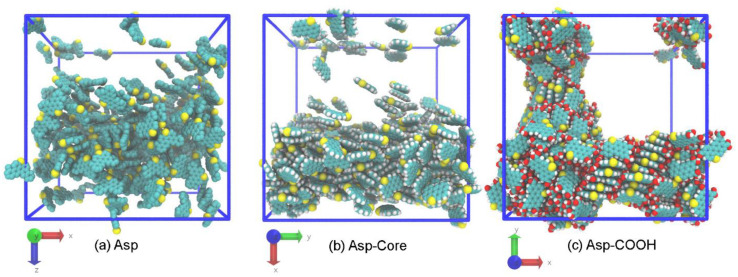
Typical snapshots of the asphaltene phase in the P3HT-based mixtures for the systems comprising (**a**) Asp, (**b**) Asp-Core and (**c**) Asp-COOH. In all snapshots, only polyaromatic cores are shown for clarity purposes.

As can be seen from the snapshot in Figure 2a, the Asp molecules remain almost uniformly distributed in the polymer phase throughout the entire simulation run, demonstrating the formation of loose aggregates. In the blends containing the Asp-Core molecules, they tend to aggregate, and phase separation is observed where the asphaltene and the polymer phase form a layered structure. However, even after the 5 µs long run, individual molecules are still present in the polymer phase (Figure 2b). Finally, the Asp-COOH molecules form a separate phase and tend to develop highly ordered stacks within it (Figure 2c). The feature of the phase separation occurring in this system is the formation of interpenetrating three-dimensional structure of the asphaltenes’ and polymer phases. The distribution of asphaltenes in the considered blends and the formation of separate phases in the Asp-Core/P3HT and Asp-COOH/P3HT mixtures are confirmed by the asphaltenes’ density distributions along the principal axes in the studied systems (Supporting Information, Figure S7.1).

The reason for the uniform distribution of Asp can be explained by the presence of aromatic and aliphatic constituents in both the asphaltenes and P3HT. A comparison of mixtures comprising the Asp-Core and Asp-COOH asphaltenes shows that the formation of the separate asphaltene phase in these systems is mostly governed by the π–π-interactions between asphaltene molecules and the absence of aliphatic groups, which lowers the affinity between the polymer and asphaltenes. However, the π–π-interactions themselves are not enough for the formation of ordered stacks by asphaltene molecules, and the interactions between the polar carboxyl groups that further decrease the affinity between the components stabilize the stacks formed by asphaltene molecules and lead to the formation of interpenetrating morphology in Asp-COOH/P3HT mixtures. Thus, the chemical nature of the side groups or their absence significantly affect the homogeneity of the P3HT/asphaltene blends even in a P3HT melt.

To estimate the miscibility and interaction strength between the components of the blends, we calculated the solubility parameter *δ* using the Hildebrand approach (see Supporting Information for details on calculations) [77,78,79]. The results obtained are collected in Table 1. The closest δ values are found for P3HT and Asp with Δ*δ* = *δ*_P3HT_ − _δASP_ equal to −0.3 (J/cm^3^)^0.5^. Both of them have aromatic and aliphatic constituents, which results in close *δ* values, visible miscibility between the compounds and no pronounced asphaltene aggregates formed. When the aliphatic side groups of the asphaltenes are cut off, the difference with the solubility parameter of P3HT increases to Δ*δ* = 3.2 (J/cm^3^)^0.5^. This can be explained by stronger interactions between the pure aromatic asphaltenes, which leads to the formation of asphaltene aggregates in the mixture with P3HT. Finally, the attachment of carboxyl groups to the polyaromatic core of the asphaltenes results in further increases in the *δ* value of asphaltenes. This means that the interaction energy between the Asp-COOH molecules is greater than that between the Asp-Core. Therefore, there should be additional interactions between Asp-COOH asphaltenes that stabilize the aggregates formed by these molecules. So, for the Asp-COOH/P3HT blends, the formation of heterophase morphology can be expected even on a macroscale. This conclusion agrees well with the observed morphological differences between the studied blends (Figure 2).

The difference between the structure of the asphaltene aggregates is drastic in the systems with aliphatic groups cut off and with carboxyl groups (see Figure 2b,c). Thus, the result suggests that the π–π interactions between the pure aromatic cores without aliphatic groups could be insufficient for the formation of ordered stacks of the asphaltenes in P3HT, and the hydrogen bonds between the –COOH groups of the adjacent asphaltenes might contribute to the stacking significantly. To estimate the role of hydrogen bonding in the structure of Asp-COOH aggregates, we estimated the number of hydrogen bonds between the Asp-COOH molecules in the blends with P3HT. To calculate the number of hydrogen bonds, the geometrical criteria were used. It was assumed that a hydroxyl group forms a hydrogen bond with a hydrogen if the distance between the donor and the acceptor oxygens is *r*_OO_ ≤ 0.35 nm, and the angle between the O–O axis and one of the O–H bonds is below 30° [80]. Typical snapshots of a system with hydrogen bonds visualized are provided in the Supporting Information, Figure S8.

The average number of H-bonds per Asp-COOH molecule was found to be 1.9. This value means that most of the -COOH groups in modified asphaltenes are involved in the formation of hydrogen bonds and confirms our conclusion on the role of hydrogen bonding in the formation of Asp-COOH stacked structures.

**Figure 3:**
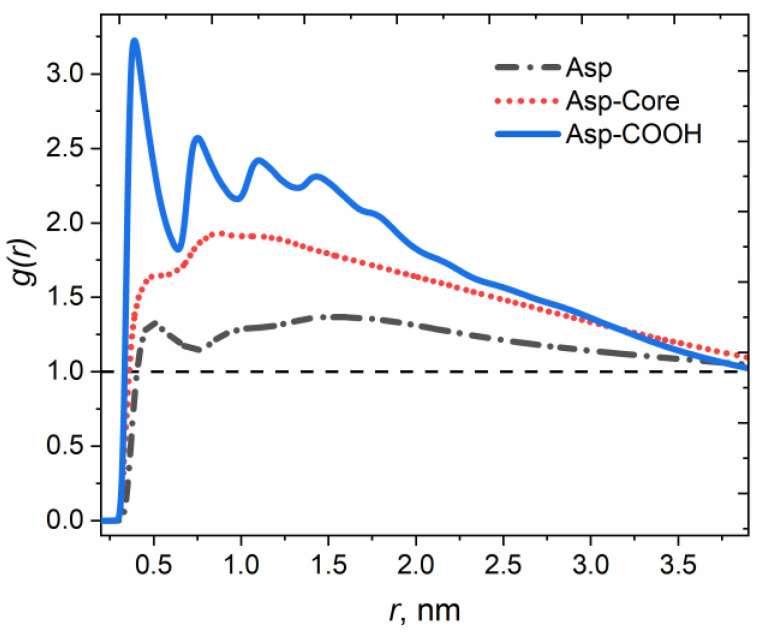
Intermolecular pair correlation functions *g(r)* between the asphaltenes calculated for the atoms in the polyaromatic cores of the asphaltene molecules (carbon and sulfur).

Figure 3 shows the intermolecular pair correlation functions *g(r)* between the asphaltenes calculated for the carbon and sulfur atoms in the polyaromatic cores of the molecules (see Supporting Information for details on calculations). The shape of the *g(r)* curves confirms that only Asp-COOH form highly ordered stacks within the aggregates, as was discussed above (see Figure 2). As can be seen from the *g(r)* curve for these asphaltenes, the distance between the peaks is approximately equal to 0.5 nm, which means that the planes of adjacent asphaltene cores are located at the distances typical for molecules stacked due to π–π interactions. It is worth noting that classical force fields do not directly take into account the quantum effects; however, being semi-empirical, they allow one to indirectly reproduce such interactions as, for example, π-π-stacking [81]. While several prominent peaks are observed for Asp-COOH, the curves for Asp and Asp-Core indicate the absence of significant ordering within the systems. Moreover, the proximity of *g(r)* for Asp to unity indirectly indicates a better miscibility between this type of particles and P3HT than that between the Asp-Core and the polymer.

**Figure 4:**
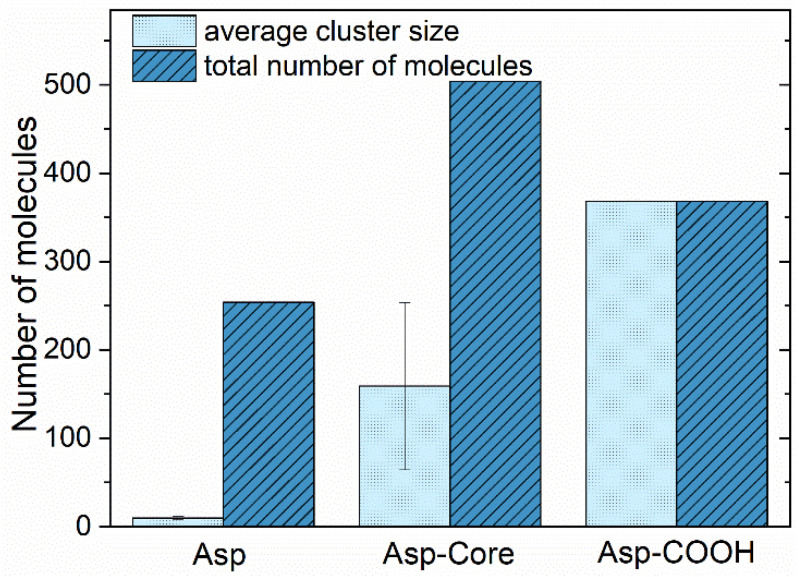
Average sizes of the asphaltene aggregates at the end of the simulations, along with the reference values of the total number of asphaltene molecules in the systems.

Figure 4 represents the column graph of the average asphaltene cluster sizes estimated over the last 100 ns of simulations that represent a well-equilibrated state of the systems used. The cluster sizes were calculated using the GROMACS *gmx clustsize* routine with the fixed cut-off distance of 0.5 nm (the largest distance between the atoms of polyaromatic cores of the asphaltene molecules was assumed to be in the same cluster) chosen on the basis of the calculated pair correlation functions (Figure 3). As seen from Figure 4, the average cluster size for Asp is equal to 10 ± 2 molecules. The Asp-Core tend to form larger aggregates with rather high variation of sizes, which, on average, are about 159 ± 95 molecules as compared to Asp. Finally, in the system with Asp-COOH, all the molecules form a single aggregate of 368 asphaltenes, which equals the total amount of molecules in the system.

The obtained computer simulation results show that in the mixtures of Asp-Core and Asp-COOH molecules with P3HT, the phase separation tends to occur, and a rather low miscibility of the components can be expected. This conclusion is also supported by the calculations of the solubility parameter that reflects the interaction energy between molecules of a certain type. Thus, we showed how the chemical modification of asphaltenes can affect the morphology of asphaltene/polythiophene mixtures, and these findings can direct further experimental development of organic solar cells utilizing asphaltenes as an acceptor. At the same time, we suppose that the miscibility of asphaltenes with polymer donors could be adjusted by both a rational choice of the donor polymer and a chemical modification of the asphaltenes.

### 3.2. Structure of Polymer Phase

Taking into account that only the crystalline domains of conjugated polymers (including P3HT) contribute to the electrical conductivity in an active layer of BHJ solar cells, the ability of carbon nanoparticles to induce the crystallization of the polymer is of high interest. So, we estimated the structure of the P3HT phase in the blends in order to find out whether the formation of ordered asphaltene aggregates can lead to the initial stages of P3HT ordering.

**Figure 5:**
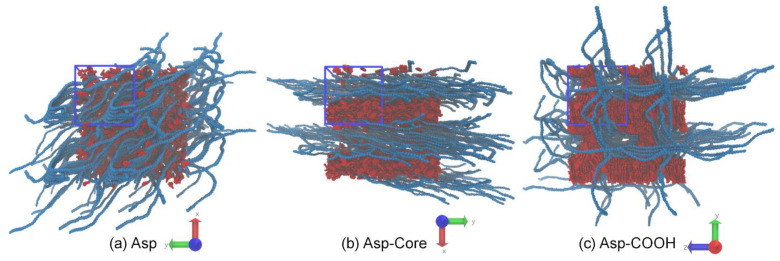
Typical snapshots of the mixture of asphaltenes (red) and P3HT (blue) obtained at the end of the 5 µs long simulation: (**a**) Asp, (**b**) Asp-Core and (**c**) Asp-COOH. Two periodic images in all directions are represented; the blue cube denotes the periodic simulation box. Aliphatic side groups are not represented for clarity.

According to the typical snapshots shown in Figure 5, P3HT chains tend to elongate in the presence of the asphaltenes. In particular, the polymer backbones are most ordered in the system comprising Asp-Core, as discussed below. In this system, the interactions between the asphaltene molecules and P3HT backbones are also mostly due to π-π stacking between the polyaromatic fragments of the molecules, not affected by the van der Waals forces between the aliphatic side groups of the molecules. Strong π–π interactions between the asphaltene cores themselves lead to the observed phase separation. Thus, the ordered structure of P3HT chains is not disturbed in this system by asphaltene molecules distributed inside the P3HT phase. Therefore, the Asp-Core molecules have a stronger impact on the polymer chain ordering, which is mainly associated with the ordering of conjugated backbones. Fragments of P3HT chains tend to form small ordered domains, reminiscent of a lamellar structure in all the systems, which are typically observed in P3HT samples [36,38,39,82]. Unfortunately, it is not possible to identify which type of P3HT polymorph could be formed in the systems considered due to the rather slow crystallization process of P3HT in comparison with the simulation timescales available and the spatial constraints observed in the systems.

**Figure 6:**
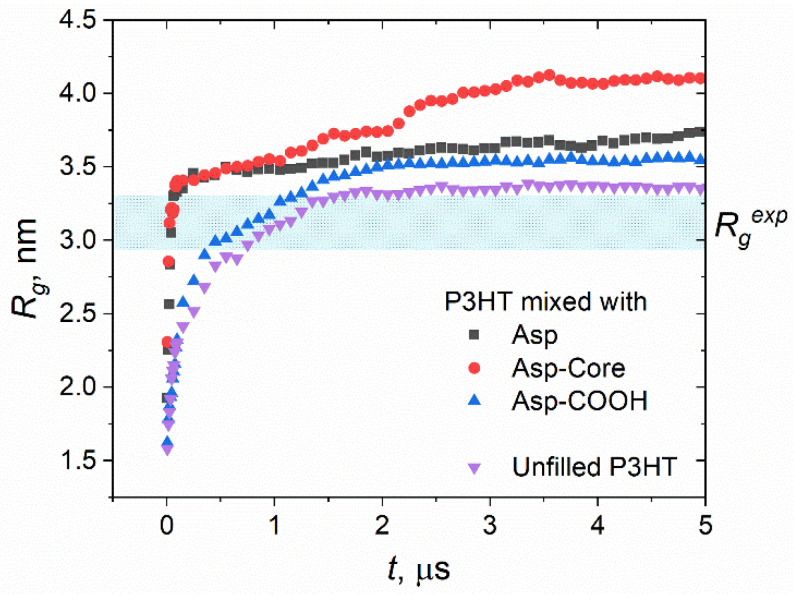
Radius of gyration *R_g_* of P3HT chains in the systems with asphaltenes and in the unfilled P3HT sample. The data on the unfilled P3HT sample were taken from our previous work [24]. Dotted blue region indicates the experimental *R_g_^exp^* values known from the literature [83,84].

The time dependence of the radius of gyration *R_g_* calculated for P3HT chains in the systems with three types of the studied asphaltenes and in an unfilled P3HT sample is represented in Figure 6. The $\sqrt{R_{g}^{2}}$ values were calculated using the GROMACS *gmx polystat* routine and then averaged over 10 ns time periods. The data on the unfilled P3HT sample containing 27 molecules were taken from our previous work for comparison purposes [24]. The simulation procedure was similar to the one described for the blends in the Models and Methods section of the present paper. It is also worth noting that the *R_g_* values for the unfilled P3HT lie at the upper boundary of the reported experimental data [83,84] obtained for the solution of P3HT in good solvents, where the polymer coils are Gaussian. The simulation results could be compared to these experimental values directly, as a polymer chain conformation in a melt also corresponds to the Gaussian coil.

First, the values reach the plateau after approximately 2.5 µs of simulations. Therefore, after this time, the simulation trajectory could be used for analysis of the blends’ structure. Second, the introduction of the asphaltenes to the polymer leads to an increase in the equilibrium *R_g_* values. The most prominent difference is observed in the case of Asp-Core, in full accordance with the behavior of P3HT observed in the snapshots (Figure 5). Thus, the presence of the asphaltene filler might lead to the initial stages of polymer ordering.

To characterize the shape and orientation of P3HT chains in the systems studied, the average values of the radius of gyration *R_g_*, the asphericity *b* and acylindricity *c* parameters, as well as the nematic ordering parameter *S_N_* [85,86], were calculated for P3HT chains over the last 2.5 µs of simulations using the following formulae:

(1) $$b=R_{g,x}^{2}-1/2\left(R_{g,y}^{2}+R_{g,z}^{2}\right)$$

(2) $$c=R_{g,y}^{2}-R_{g,z}^{2}$$

where *R_g,α_ (α* ∊ *{x,y,z})* are the eigenvalues of the chain inertia tensor [87]. The results obtained are provided in Table 2.

The explanation for the results observed could be as follows. The Asp molecules are too miscible with P3HT. Therefore, the blends formed are rather uniform, and only slight elongation of the polymer chains is observed. However, the P3HT chains are more oriented in the blends with Asp in comparison with unfilled P3HT. At the same time, the Asp-COOH form dense aggregates, leaving too little free space for the polymer chains to elongate and form a structure where all chains are ordered in the same direction. Thus, in the Asp-COOH/P3HT blends, the order parameter is much lower than that in blends with Asp and Asp-Core, although the shape of the P3HT chain is close to cylindrical, as follows from the values of asphericity and acylindricity. In the system with the Asp-Core, phase separation occurs with the formation of a layered structure (see density profiles in Supporting Information, Figure S7.1). The P3HT chains stretch and order within the flat layer bounded by the asphaltene phase. Thus, the Asp-Core phase serves as a director defining the orientation of the polymer chains most likely due to the π–π interactions between the polyaromatic core of the asphaltenes and the aromatic polymer backbone unbothered by the aliphatic side groups surrounding the asphaltene core.

### 3.3. Concentration Effects

As was shown in the previous paragraph, the introduction of the asphaltenes to the polymer matrix can indeed lead to the ordering of the polymer chains. However, in the system with Asp-COOH, possessing the most promising electronic properties [24], the too large and dense cluster prevents the elongation of the polymer chains. As the crystalline domains of the polymer are responsible for the charge transfer in the donor phase, an increase in the degree of crystallinity is highly desirable. The question arises, namely, can we influence the structure of the blends and ordering of P3HT chains by changing the concentration of Asp-COOH, thus moderating the size of the aggregates?

**Figure 7:**
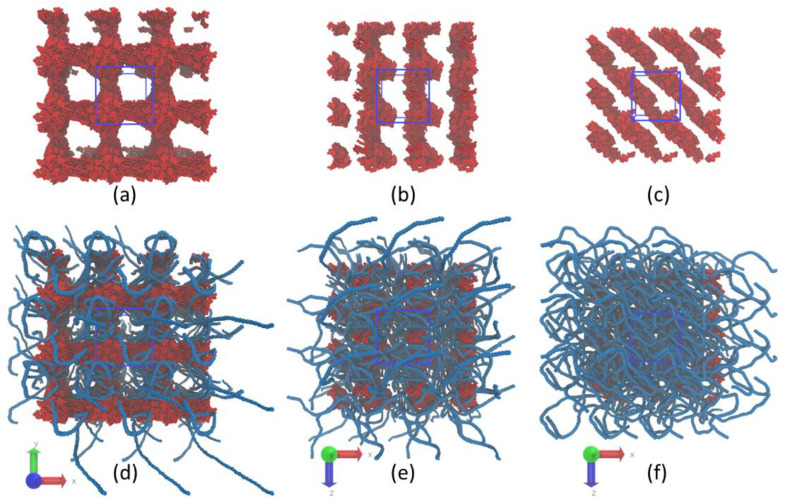
Typical snapshots of the (**a**–**c**) Asp-COOH phase and (**d**–**f**) Asp-COOH/P3HT blends at concentrations equal to (**a**,**d**) 1:1, (**b**,**e**) 2:3 and (**c**,**f**) 1:3 obtained at the end of the 5 µs long simulations. The top row shows only asphaltene molecules, while the bottom row also represents the backbones of the P3HT chains. Several periodic images are represented in each snapshot. The blue cube denotes the periodic simulation box. Aliphatic side groups are not represented for clarity.

As can be seen from snapshots in Figure 7, already with a decrease in the asphaltenes/P3HT ratio from 1:1 to 2:3, the three-dimensional structure of the asphaltene aggregates is disrupted, and elongated linear aggregates are observed. The analysis of the asphaltene density distributions along the principal axes (Supporting Information, Figure S7.2) shows that in the blends with asphaltenes/P3HT ratio equal to 2:3 and 1:3, the asphaltene phase forms columnar structures. Clearly, the concentration of the asphaltenes becomes too low to form the interpenetrating three-dimensional blend morphology.

The results on the size, shape and orientation of P3HT chains provided in Table 2 demonstrate that the decrease in the concentration of asphaltenes in the system leads to a decrease in asphericity *b* and an increase in acylindricity *c*. Thus, the interplay between the parameters *b* and *c* indicates that the polymer chains approach the coil conformation in the mixtures with a decreasing amount of asphaltenes. Moreover, the comparison of the gyration radii of the polymer chains in the unfilled sample and the studied blends suggests that, at the minimum investigated concentration of the asphaltenes, the polymer coils are even denser than in the case of the unfilled P3HT sample. Our assumption is that, at a low content of asphaltenes in the system, the polymer chains tend to wrap up around the elongated linear aggregate of the asphaltenes (snapshot is provided in Supporting Information, Figure S9), i.e., Asp-COOH at small concentrations act as adsorbents, attracting P3HT aromatic backbones and preventing the formation of ordered structures. Since both the elongation of the polymer chains and the formation of the interpenetrating 3D structure of the two phases are required to obtain an efficient active layer of BHJ solar cells, the concentration of asphaltenes/P3HT mixtures should be at least 1:1. However, we can assume that with a further decrease in P3HT concentration, the interpenetrating three-dimensional structure of the P3HT phase will be destroyed, as was observed for the asphaltenes at low concentrations (2:3 and 1:3).

## 4. Conclusions

In this work, we addressed the effect of the chemical structure of the asphaltenes on their aggregation behavior in mixtures with P3HT and on the morphology of the polymer phase, including the possibility of the formation of the interpenetrating 3D structure within the polymer and asphaltene phases at varying concentrations of carboxyl-containing asphaltenes that could be essential for the blends intended for use in BHJ solar cells.

It was found that the chemical structure of asphaltenes and their interaction with P3HT polymer chains play a crucial role in the morphology of the blends studied. Asphaltenes with aliphatic side groups were almost evenly distributed in P3HT, whereas asphaltenes with aliphatic groups cut off formed a loose cluster, and two distinct non-interpenetrating phases were formed in this composite, while the carboxyl-containing asphaltenes aggregated with the formation of prominent stacked structures. A comparison of the aggregation behavior of asphaltenes revealed that the formation of stacks was due to the π–π interactions between the polyaromatic cores of the asphaltenes, additionally stabilized by hydrogen bonds.

It was shown that the asphaltene phase can serve as a director defining the orientation of the polymer chains and affect the ordering of the chains due to the π–π interactions between the polyaromatic core of the asphaltenes and the aromatic polymer backbone if the interaction between the polymer chains and the asphaltene nanoparticles is unbothered by the aliphatic side groups surrounding the asphaltene core.

Thus, the present work reveals the impact of the asphaltenes’ chemical composition and concentration on the structure of the asphaltenes/P3HT blends, which may contribute to the development of low-cost BHJ solar cells based on conjugated polymer/asphaltene blends. To continue our investigation further, a collaboration could be established with experimental groups, which specialize in asphaltene extraction from natural sources and modification of asphaltenes, as well as investigation of organic photovoltaic devices. We believe that our results will help with finding a proper method of asphaltene treatment and mixture composition to develop new organic solar cells with improved performance and reduced production costs.

## Figures and Tables

**Table 1:** Estimated solubility parameter *δ*.

Sample	*δ*, (J/cm^3^)^0.5^
P3HT	12.6
Asp	12.3
Asp-Core	15.8
Asp-COOH	26.7

**Table 2:** Equilibrium values of the radius of gyration *R_g_*, asphericity *b* and acylindricity *c* parameters, nematic order parameter *S_N_* obtained for the P3HT chains in the unfilled sample [24] and in the blends with Asp-COOH at concentrations of 1:1, 2:3 and 1:3.

System		*R_g_,* nm	*b*	*c*	*S_N_*
Asp-COOH/P3HT	1:1	3.53 ± 0.03	4.0 ± 0.4	0.6 ± 0.3	0.22 ± 0.03
	2:3	3.3 ± 0.1	1.9 ± 0.3	2.1 ± 0.2	0.17 ± 0.04
	1:3	3.04 ± 0.04	2.4 ± 0.2	3.2 ± 0.2	0.26 ± 0.05
Asp/P3HT		3.6 ± 0.1	8.2 ± 1.1	2.6 ± 0.6	0.66 ± 0.08
Asp-Core/P3HT		4.0 ± 0.1	11.6 ± 3.5	2.1 ± 1.4	0.7 ± 0.2
Unfilled P3HT		3.35 ± 0.03	4.2 ± 0.3	3.0 ± 0.3	0.43 ± 0.04

## Data Availability

Not applicable.

## Supplementary Materials

The supporting information can be downloaded at: https://www.mdpi.com/article/10.3390/nano12162867/s1, containing force field parameters for P3HT and the asphaltenes, estimation of simulation times required for the system equilibration, asphaltene phase density profiles, calculation of solubility parameter *δ*, data on hydrogen bonding in the systems studied, calculation of intermolecular pair correlation function *g(r)*.
