# Supplementary material for: Influence of Asphaltene Modification on Structure of P3HT/Asphaltene Blends: Molecular Dynamics Simulations
Source: Nanomaterials (Basel). 2022 Aug 20;12(16):2867. doi: 10.3390/nano12162867 (PMC9413297; doi:10.3390/nano12162867)
Supplement: Supplementary file 1 [file nanomaterials-12-02867-s001.zip › nanomaterials-1850520-supplementary.pdf]

Supporting Information for

Influence of Asphaltene Modification on Structure of P3HT/Asphaltene Blends: Molecular Dynamics Simulations

Natalia Borzdun [1], Artyom Glova [1], Sergey Larin [1,2,*] and Sergey Lyulin [1]

[1] Institute of Macromolecular Compounds, Russian Academy of Sciences, Bolshoi pr. 31 (V.O.), 199004 St. Petersburg, Russia
[2] Faculty of Physics, St. Petersburg State University, Ulyanovskaya str. 1–3, Peterhof, 198504 St. Petersburg, Russia
* Correspondence: selarin@macro.ru

Force field parameters for P3HT and the asphaltenes

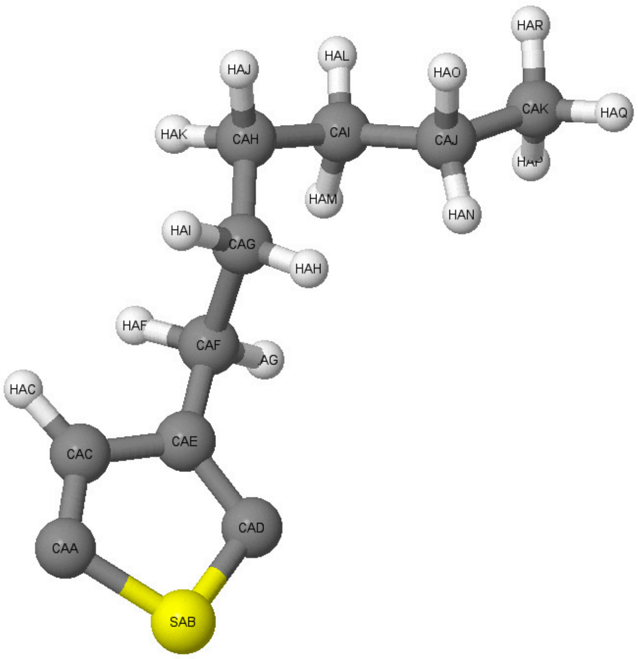

Figure S1. Structure of the P3HT monomer unit. Atom names are shown for each particle.

Table S1. GAFF atom types and partial charges of a P3HT monomer unit.

Atom name	GAFF atom type	Partial charge	Atom name	GAFF atom type	Partial charge	Atom name	GAFF atom type	Partial charge
HAP	hc	0.0328	CAI	c3	-0.0781	HAG	hc	0.0548
HAQ	hc	0.0328	HAM	hc	0.0398	CAF	c3	-0.0220
HAR	hc	0.0328	HAK	hc	0.0408	HAF	hc	0.0548
CAK	c3	-0.0928	CAH	c3	-0.0791	CAE	cc	-0.0780
HAO	hc	0.0378	HAJ	hc	0.0408	CAC	cc	-0.1540
CAJ	c3	-0.0791	HAH	hc	0.0423	HAC	ha	0.1580
HAN	hc	0.0378	CAG	c3	-0.0741	CAA	cd	-0.0250
HAL	hc	0.0398	HAI	hc	0.0423	SAB	ss	0.0363
						CAD	cd	-0.0420

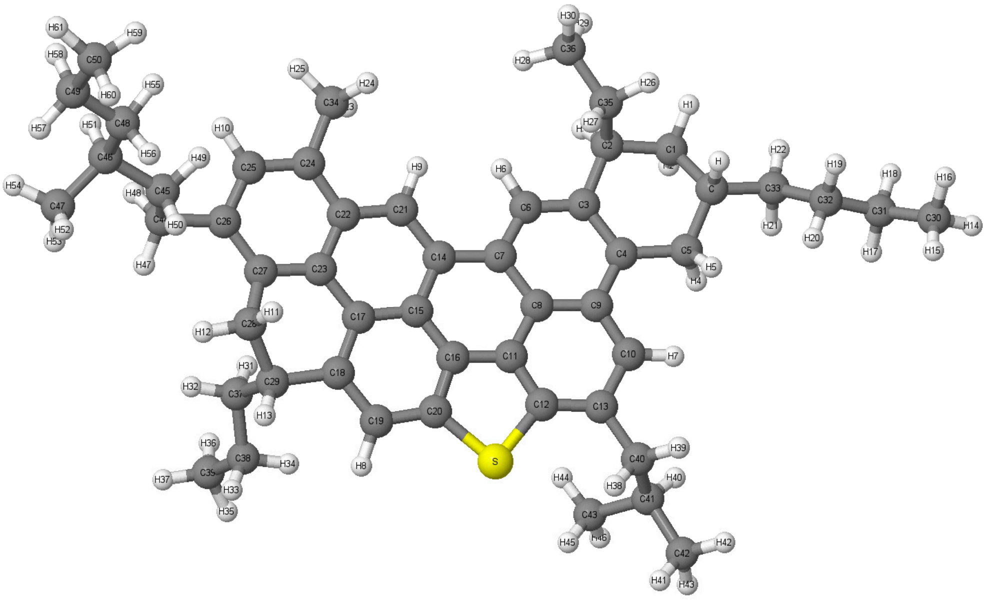

Figure S2. Structure of the asphaltene with aliphatic side groups (Asp). Atom names are shown for each particle.

Table S2. GAFF atom types and partial charges of the asphaltene with aliphatic side groups (Asp).

Atom name	GAFF atom type	Partial charge	Atom name	GAFF atom type	Partial charge	Atom name	GAFF atom type	Partial charge
C	c3	-0.0617	C27	ca	-0.0643	H36	hc	0.034033
H	hc	0.0547	C28	c3	-0.0331	H37	hc	0.034033
C1	c3	-0.0694	H11	hc	0.0577	C40	c3	-0.0371
H1	hc	0.0447	H12	hc	0.0577	H38	hc	0.0517
H2	hc	0.0447	C29	c3	-0.0174	H39	hc	0.0517
C2	c3	-0.0184	H13	hc	0.0647	C41	c3	-0.0627
H3	hc	0.0577	C30	c3	-0.0931	H40	hc	0.0487
C3	ca	-0.0713	H14	hc	0.0330	C42	c3	-0.0926
C4	ca	-0.0593	H15	hc	0.0330	H41	hc	0.0362
C5	c3	-0.0341	H16	hc	0.0330	H42	hc	0.0362
H4	hc	0.0522	C31	c3	-0.0804	H43	hc	0.0362
H5	hc	0.0522	H17	hc	0.0382	C43	c3	-0.0926
C6	ca	-0.1060	H18	hc	0.0382	H44	hc	0.0362
H6	ha	0.1350	C32	c3	-0.0804	H45	hc	0.0362
C7	ca	-0.0160	H19	hc	0.0417	H46	hc	0.0362
C8	ca	0.0030	H20	hc	0.0417	C44	c3	-0.0371
C9	ca	-0.0170	C33	c3	-0.0764	H47	hc	0.0487
C10	ca	-0.1150	H21	hc	0.0402	H48	hc	0.0487
H7	ha	0.1340	H22	hc	0.0402	C45	c3	-0.0764
C11	ca	-0.0570	C34	c3	-0.0558	H49	hc	0.0467
C12	ca	-0.0441	H23	hc	0.0450	H50	hc	0.0467
C13	ca	-0.0283	H24	hc	0.0450	C46	c3	-0.0667
C14	ca	-0.0090	H25	hc	0.0450	H51	hc	0.0477

Atom name	GAFF atom type	Partial charge	Atom name	GAFF atom type	Partial charge	Atom name	GAFF atom type	Partial charge
C15	ca	0.0110	C35	c3	-0.0774	C47	c3	-0.0901
C16	ca	-0.0640	H26	hc	0.0427	H52	hc	0.034367
C17	ca	-0.0200	H27	hc	0.0427	H53	hc	0.034367
C18	ca	-0.0463	C36	c3	-0.0941	H54	hc	0.034367
C19	ca	-0.0900	H28	hc	0.034367	C48	c3	-0.0774
H8	ha	0.1370	H29	hc	0.034367	H55	hc	0.0397
C20	ca	-0.0451	H30	hc	0.034367	H56	hc	0.0397
C21	ca	-0.1030	C37	c3	-0.0774	C49	c3	-0.0804
H9	ha	0.1340	H31	hc	0.0457	H57	hc	0.0397
C22	ca	-0.0310	H32	hc	0.0457	H58	hc	0.0397
C23	ca	-0.0120	C38	c3	-0.0824	C50	c3	-0.0931
C24	ca	-0.0663	H33	hc	0.0407	H59	hc	0.0327
C25	ca	-0.1250	H34	hc	0.0407	H60	hc	0.0327
H10	ha	0.1310	C39	c3	-0.0941	H61	hc	0.0327
C26	ca	-0.0673	H35	hc	0.034033	S	ss	0.0322

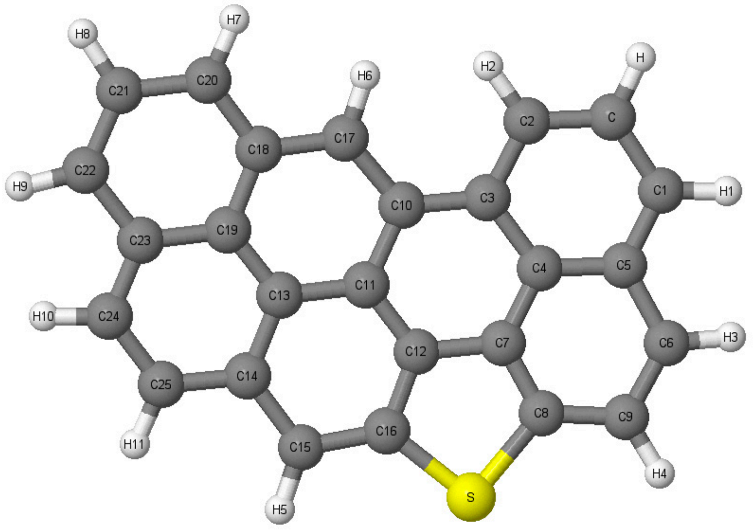

Figure S3. Structure of the asphaltene with aliphatic side groups cut off (Asp-Core). Atom names are shown for each particle.

Table S3. GAFF atom types and partial charges of the asphaltene with aliphatic side groups cut off (Asp-Core).

Atom name	GAFF atom type	Partial charge	Atom name	GAFF atom type	Partial charge	Atom name	GAFF atom type	Partial charge
C	ca	-0.1310	C9	ca	-0.0850	C19	ca	-0.0130
H	ha	0.1330	H4	ha	0.1390	C20	ca	-0.1110
C1	ca	-0.1070	C10	ca	-0.0090	H7	ha	0.1340
H1	ha	0.1350	C11	ca	0.0100	C21	ca	-0.1290
C2	ca	-0.1100	C12	ca	-0.0580	H8	ha	0.1340
H2	ha	0.1330	C13	ca	-0.0170	C22	ca	-0.1090
C3	ca	-0.0140	C14	ca	-0.0230	H9	ha	0.1350
C4	ca	0.0070	C15	ca	-0.0730	C23	ca	-0.0340
C5	ca	-0.0360	H5	ha	0.1430	C24	ca	-0.1110
C6	ca	-0.1080	C16	ca	-0.0351	H10	ha	0.1340
H3	ha	0.1340	C17	ca	-0.0980	C25	ca	-0.1160
C7	ca	-0.0630	H6	ha	0.1350	H11	ha	0.1340
C8	ca	-0.0371	C18	ca	-0.0330	S	ss	0.0202

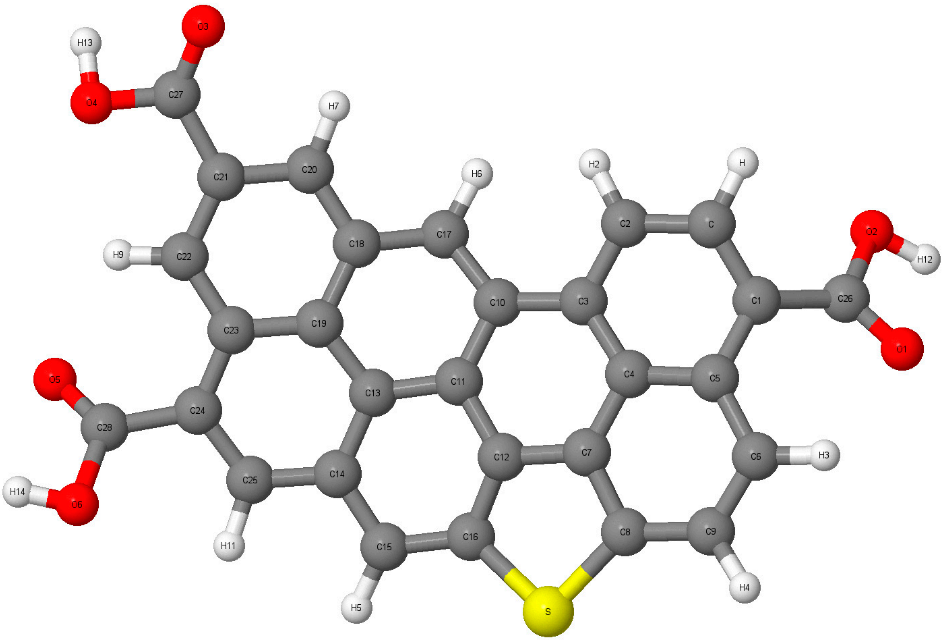

Figure S4. Structure of the modified asphaltene with carboxyl groups (Asp-COOH). Atom names are shown for each particle.

Table S4. GAFF atom types and partial charges of the modified asphaltene with carboxyl groups (Asp-COOH).

Atom name	GAFF atom type	Partial charge	Atom name	GAFF atom type	Partial charge	Atom name	GAFF atom type	Partial charge
C	ca	-0.065000	C12	ca	-0.048000	C24	ca	-0.106600
H	ha	0.159000	C13	ca	-0.014000	C25	ca	-0.034000
C1	ca	-0.111600	C14	ca	-0.044000	H11	ha	0.159000
C2	ca	-0.131000	C15	ca	-0.054000	S	ss	0.052200
H2	ha	0.144000	H5	ha	0.147000	C26	c	0.649702
C3	ca	0.021000	C16	ca	-0.044100	O1	o	-0.550001
C4	ca	-0.006000	C17	ca	-0.076000	O2	oh	-0.604101
C5	ca	0.014000	H6	ha	0.143000	H12	ho	0.448000
C6	ca	-0.104000	C18	ca	-0.056000	C27	c	0.650702
H3	ha	0.161000	C19	ca	0.013000	O3	o	-0.554001
C7	ca	-0.067000	C20	ca	-0.049000	O4	oh	-0.597101
C8	ca	-0.039100	H7	ha	0.162000	H13	ho	0.449000
C9	ca	-0.079000	C21	ca	-0.132600	C28	c	0.650702
H4	ha	0.145000	C22	ca	-0.045000	O5	o	-0.545001
C10	ca	-0.024000	H9	ha	0.192000	O6	oh	-0.612101
C11	ca	0.014000	C23	ca	-0.032000	H14	ho	0.450000

Estimation of simulation times required for the system equilibration

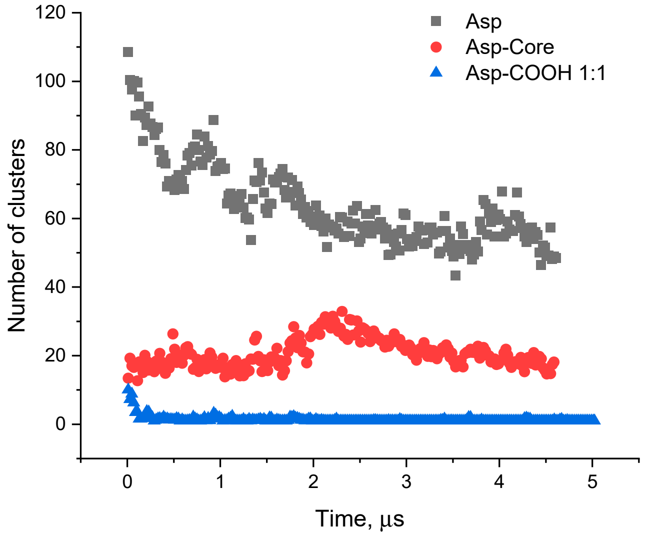

Figure S5. Number of asphaltene clusters in the studied blends (P3HT/Asp, P3HT/Asp-Core, P3HT/Asp-COOH at a concentration of 1:1) as a function of time.

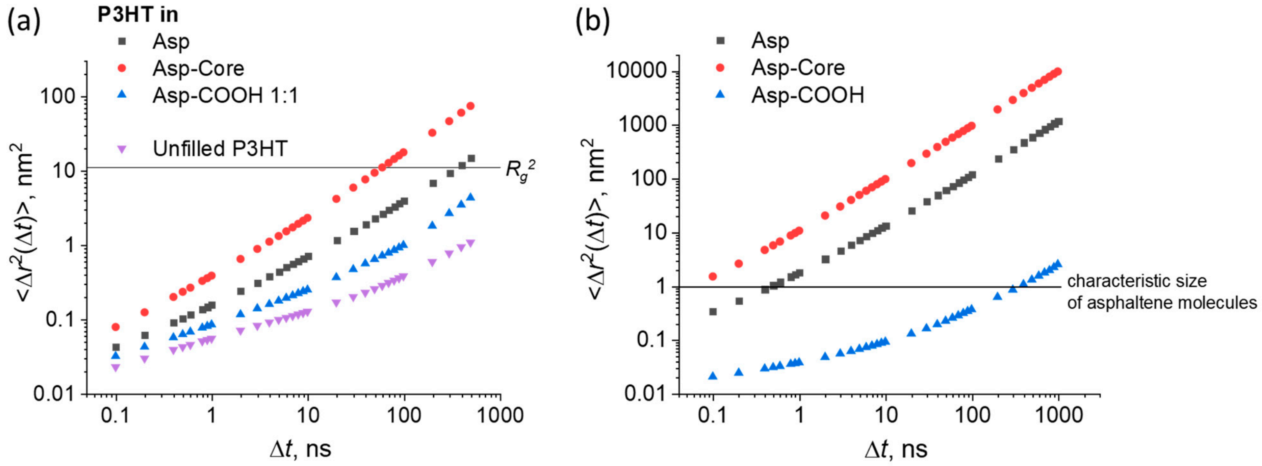

Figure S6. Mean-squared displacements of the centers of mass of (a) the P3HT chains and (b) the asphaltenes in the studied systems. Horizontal lines represent the squared gyration radius of P3HT and characteristic sizes of the asphaltene molecules.

Asphaltene phase density profiles

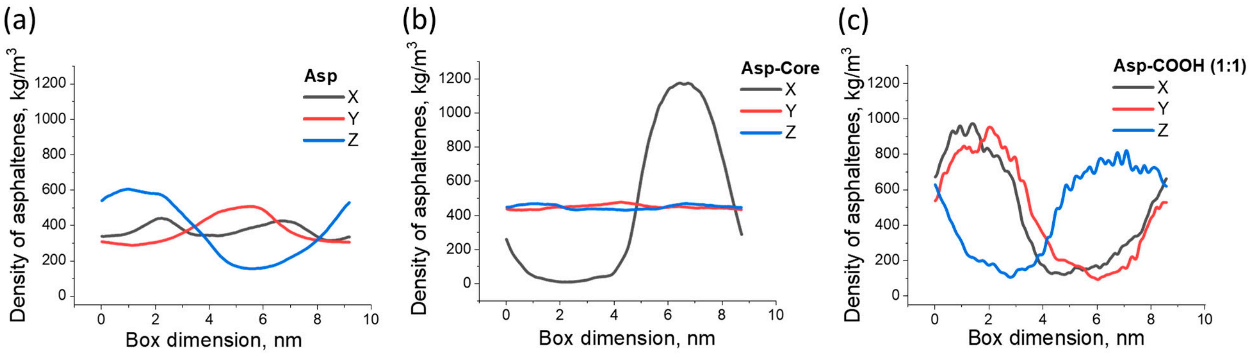

Figure S7.1. Density profiles of the asphaltene phase along the X, Y and Z axes obtained for the following blends by averaging over the last 100 ns of simulations: (a) P3HT/Asp, (b) P3HT/Asp-Core and (c) P3HT/Asp-COOH (at a concentration of 1:1).

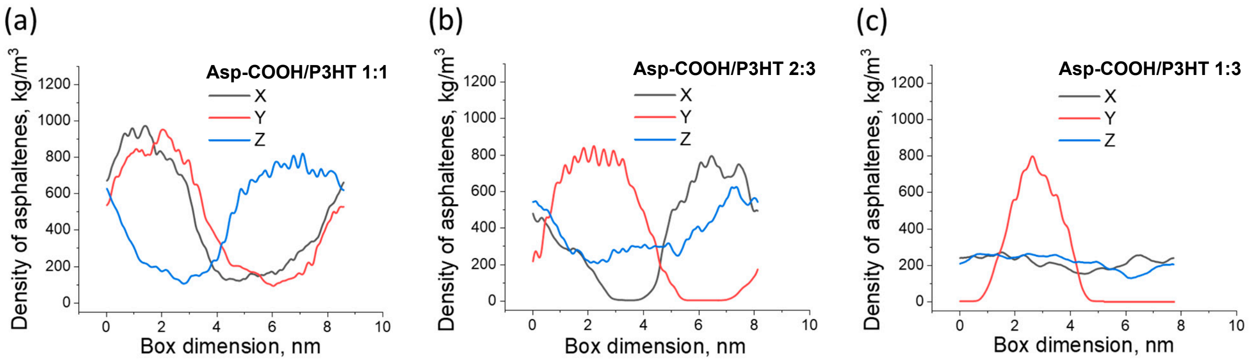

Figure S7.2. Density profiles of the asphaltene phase along the X, Y and Z axes obtained by averaging over the last 100 ns of simulations of Asp-COOH/P3HT blends at concentrations equal to: (a) 1:1, (b) 2:3 and (c) 1:3.

Calculation of solubility parameter δ

The solubility parameter δ was calculated using the following formula:[1]

$$\delta_i = \sqrt{\frac{E_{coh}}{V_m}},$$

where $\frac{E_{coh}}{V_m}$ is the cohesive energy density, E_{coh} is the intermolecular interaction energy, V_m is the system molar volume.

E_{coh} in turn is defined as follows:[2]

$$E_{coh} = \sum_{N} E_{is} - E_{tot},$$

where N is the number of polymer chains, E_{is} is the potential energy of individual chains, E_{tot} is the total potential energy of the system.

The potential energy of an individual chain E_{is} was obtained as follows. First, the MD trajectories were converted into separate trajectories corresponding to the individual chain in a simulation box using the GROMACS *gmx mdun –rerun* routine. Then, the time dependencies of the potential energies of isolated chains E_{is} and the total potential energy of the system were evaluated with the aid of the *gmx energy* routine. As a result, the time dependencies of E_{coh} and the solubility parameter δ were obtained, according to the equations above.

Hydrogen bonding

We utilized the GROMACS *gmx hbond* routine to calculate the average number of hydrogen bonds in the P3HT/Asp-COOH blend. The value obtained for the last 100 ns of 5 μs long simulation was equal to 1.9 hydrogen bonds per Asp-COOH molecule.

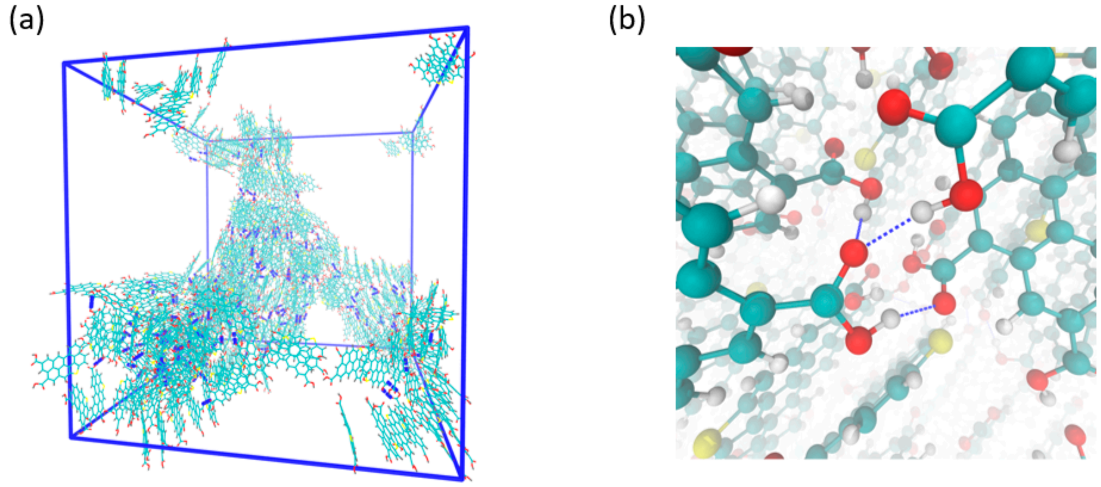

Figure S8. (a) Typical snapshot of the Asp-COOH phase obtained at the end of the 5 μs long simulation. Hydrogen bonds are represented as blue lines. (b) Close-up view of the possible pathways for the formation of hydrogen bonds between carboxyl groups of the asphaltenes from one stack and from adjacent stacks. Carbon, oxygen and hydrogen atoms are represented in cyan, red and light-gray, respectively.

Calculation of intermolecular pair correlation function g(r)

Intermolecular pair correlation function *g(r)* between the atoms in the polyaromatic cores of the asphaltene molecules (carbon and sulfur) was calculated using the following equation:[3]

$$g(r) = \frac{\langle \rho(r) \rangle}{\langle \rho \rangle_{local}} = \frac{1}{\langle \rho \rangle_{local}} \frac{1}{N} \sum_{i=1}^{N} \frac{\delta(r_i - r)}{4\pi r^2},$$

Here, $\langle \rho(r) \rangle$ is the average number density of the asphaltene atoms at the distance r from the given asphaltene atoms; $\langle \rho \rangle_{local}$ is the average number density of the asphaltenes in the system; N is the number of asphaltenes; r_i is the distance between the pair of asphaltenes, and δ is the Kronecker delta.

P3HT fragment wrapping around the asphaltene cluster

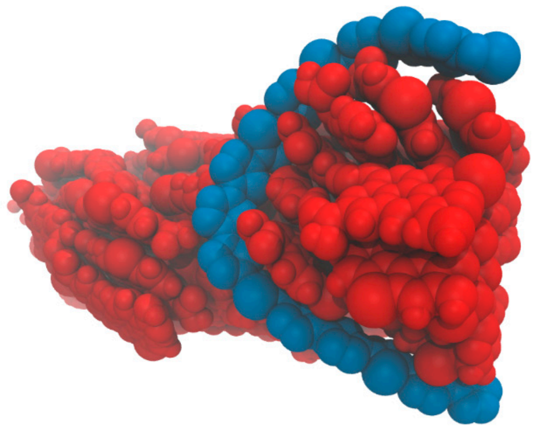

Figure S9. Typical snapshot of a fragment of the P3HT chain backbone wrapped around an Asp-COOH cluster obtained after 5 μs long simulation run for the system with lowest concentration of asphaltenes (Asp-COOH/P3HT 1:3).

References

(1) Thomas, S.; Grohens, Y.; Jyotishkumar, P. *Characterization of Polymer Blends*; Thomas, S., Grohens, Y., Jyotishkumar, P., Eds.; Wiley-VCH Verlag GmbH & Co. KGaA: Weinheim, Germany, **2014**; Vol. 9783527331. https://doi.org/10.1002/9783527645602.

(2) Belmares, M.; Blanco, M.; Goddard, W. A.; Ross, R. B.; Caldwell, G.; Chou, S. H.; Pham, J.; Olofson, P. M.; Thomas, C. Hildebrand and Hansen Solubility Parameters from Molecular Dynamics with Applications to Electronic Nose Polymer Sensors. *J. Comput. Chem.* **2004**, *25* (15), 1814–1826. https://doi.org/10.1002/JCC.20098.

(3) Larin, S. V.; Falkovich, S. G.; Nazarychev, V. M.; Gurtovenko, A. a.; Lyulin, A. V.; Lyulin, S. V. Molecular-Dynamics Simulation of Polyimide Matrix Pre-Crystallization near the Surface of a Single-Walled Carbon Nanotube. *RSC Adv.* **2014**, *4* (2), 830. https://doi.org/10.1039/c3ra45010d.
